# Association of Trimethylamine *N*-Oxide and Related Metabolites in Plasma and Incident Type 2 Diabetes: The Cardiovascular Health Study

**DOI:** 10.1001/jamanetworkopen.2021.22844

**Published:** 2021-08-27

**Authors:** Rozenn N. Lemaitre, Paul N. Jensen, Zeneng Wang, Amanda M. Fretts, Barbara McKnight, Ina Nemet, Mary L. Biggs, Nona Sotoodehnia, Marcia C. de Oliveira Otto, Bruce M. Psaty, David S. Siscovick, Stanley L. Hazen, Dariush Mozaffarian

**Affiliations:** 1 Cardiovascular Health Research Unit, Department of Medicine, University of Washington, Seattle; 2 Department of Cardiovascular and Metabolic Sciences, Lerner Research Institute, Cleveland Clinic, Cleveland, Ohio; 3 Department of Epidemiology, University of Washington, Seattle; 4 Department of Biostatistics, University of Washington, Seattle; 5 Division of Cardiology, University of Washington, Seattle; 6 Division of Epidemiology, Human Genetics and Environmental Science, School of Public Health, The University of Texas Health Science Center at Houston; 7 Kaiser Permanente Washington Health Research Institute, Seattle; 8 New York Academy of Medicine, New York, New York; 9 Department of Cardiovascular Medicine, Heart, Vascular and Thoracic Institute, Cleveland Clinic, Cleveland, Ohio; 10 Friedman School of Nutrition Science and Policy, Tufts University, Boston, Massachusetts

## Abstract

**Importance:**

Although rodent studies suggest that trimethylamine *N*-oxide (TMAO) influences glucose homeostasis and risk of type 2 diabetes, evidence in humans is limited.

**Objective:**

To examine the associations of serial measures of plasma TMAO and related metabolite concentrations with incident type 2 diabetes, fasting plasma insulin and glucose levels, and the Gutt insulin sensitivity index (ISI).

**Design, Setting, and Participants:**

This prospective cohort design assessed the association of plasma TMAO and related metabolite concentrations with diabetes outcome, whereas a cross-sectional design assessed the association with insulin and glucose levels and Gutt ISI. The participants were a cohort of older US adults from the Cardiovascular Health Study (CHS). Data from June 1989 to May 1990, from November 1992 to June 1993, and from June 1995 to June 1997 were included, with follow-up through June 2010. Levels of TMAO and related metabolites were measured in CHS plasma samples. Data were analyzed from July 2019 to September 2020.

**Exposures:**

Plasma concentrations of TMAO, carnitine, betaine, choline, crotonobetaine, and γ-butyrobetaine, measured by high-performance liquid chromatography and mass spectrometry.

**Main Outcomes and Measures:**

Linear regression for associations of TMAO and related metabolites with insulin and glucose levels and Gutt ISI, and proportional hazards regression for associations with diabetes.

**Results:**

The study included 4442 participants without diabetes at baseline (mean [SD] age, 73 [6] years at entry; 2710 [61%] women). In multivariable analyses, plasma TMAO, carnitine, crotonobetaine, and γ-butyrobetaine concentrations were positively associated with fasting insulin level (insulin mean geometric ratio comparing fifth with first quintiles of metabolite concentration: 1.07 [95% CI, 1.04-1.10] for TMAO; 1.07 [95% CI, 1.03-1.10] for carnitine; 1.05 [95% CI, 1.02-1.08] for crotonobetaine; and 1.06 [95% CI, 1.02-1.09] for γ-butyrobetaine). In contrast, betaine and choline concentrations were associated with greater insulin sensitivity (mean difference in Gutt ISI comparing fifth with first quintiles: 6.46 [95% CI, 4.32-8.60] and 2.27 [95% CI, 0.16-4.38], respectively). Incident diabetes was identified in 661 participants during a median 12.1 (interquartile range, 6.9-17.1) years of follow-up. In multivariable analyses, TMAO and metabolites were not significantly associated with type 2 diabetes risk (hazard ratios of diabetes comparing fifth with first quintile: 1.20 [95% CI, 0.94-1.55] for TMAO; 0.96 [95% CI, 0.74-1.24] for choline; 0.88 [95% CI, 0.67-1.15] for betaine; 1.07 [95% CI, 0.83-1.37] for carnitine; 0.79 [95% CI, 0.60-1.04] for γ-butyrobetaine; and 1.06 [95% CI, 0.83-1.35] for crotonobetaine).

**Conclusions and Relevance:**

Plasma TMAO and related metabolites were not significantly associated with type 2 diabetes among older adults. The metabolites TMAO, carnitine, γ-butyrobetaine, and crotonobetaine may be associated with insulin resistance, and betaine and choline may be associated with greater insulin sensitivity, but temporality of the associations was not established.

## Key Points

**Question:**

What is the association of trimethylamine *N*-oxide (TMAO) and related metabolites with the risk of type 2 diabetes?

**Findings:**

In this cohort study that included 4442 older adults, plasma concentrations of TMAO and related metabolites were not associated with the risk of incident type 2 diabetes. However, elevated concentrations of TMAO, carnitine, crotonobetaine, and γ-butyrobetaine were each cross-sectionally associated with increased fasting insulin levels, a marker of insulin resistance, whereas betaine and choline were associated with decreased fasting insulin levels and an increased index of insulin sensitivity.

**Meaning:**

Although replication is needed, these findings suggest the possibility of early involvement of TMAO metabolites with glucose-insulin homeostasis, which is detrimental for TMAO and carnitine and favorable for betaine and choline.

## Teaser

This cohort study assesses the associations of trimethylamine *N*-oxide and related metabolites with incident type 2 diabetes and its early markers in older US adults in the Cardiovascular Health Study.

## Introduction

Type 2 diabetes has reached epidemic proportions worldwide, with the number of adults having the disease doubling since 1980.^1^ Although a range of factors influence the risk of type 2 diabetes (hereafter referred to as diabetes), strong evidence suggests that diabetes is largely attributable to lifestyle factors, including poor diet.^2^ Understanding novel mechanisms that link diet to diabetes are critical.

Trimethylamine *N*-oxide (TMAO) is a metabolite derived predominantly from dietary precursors that include carnitine (primarily from red meat)^3,4^ and choline (primarily from eggs and other animal products)^5,6^ and from other dietary precursors such as phosphatidylcholine (also called lecithin), betaine (primarily from shellfish, wheat germ or bran, and spinach),^7^ trimethyllysine,^8^ and, to a lesser extent, carnitine-related compounds such as crotonobetaine and γ-butyrobetaine.^9^ In the intestine, microorganisms release trimethylamine (TMA) from these precursors. The TMA is then absorbed and metabolized into TMAO by the host hepatic flavin monooxygenases, primarily *FMO3*.^5,10^ Therefore, circulating TMAO levels are an integrated measure of diet, microbiome, and host physiology. In experimental studies, TMAO influences multiple atherosclerotic pathways^5,11,12,13^; in clinical samples of patients with prevalent risk factors, it is associated with risk of cardiovascular disease events.^6,14,15,16^

Studies in rodents have suggested TMAO could also influence glucose homeostasis and diabetes.^17,18,19,20^ However, evidence in humans is limited. In cross-sectional and case-control studies, TMAO levels are higher in patients with prevalent diabetes than those without.^21,22,23,24^ However, prospective studies do not suggest an increase in diabetes risk with elevated TMAO levels.^25,26^ Therefore, a potential influence of TMAO on the risk of incident diabetes in humans remains to be established. To address these questions, we prospectively measured plasma levels of TMAO and its related metabolites carnitine, betaine, choline, crotonobetaine, and γ-butyrobetaine in the Cardiovascular Health Study (CHS), a community-based cohort of older adults in the US. We investigated the associations of these metabolites with incident diabetes and with early markers of diabetes, including fasting plasma insulin and glucose levels and the Gutt insulin sensitivity index (ISI), a measure of insulin sensitivity.^27^

## Methods

### Study Population

Participants in the CHS were recruited from 4 US communities as described previously.^28^ The cohort consists of 5201 community-dwelling men and women who were 65 years or older at enrollment and were recruited from June 1989 to May 1990, plus an additional 687 predominantly Black participants who were recruited from November 1992 to June 1993. Race was classified by self-identification and was assessed to investigate potential differences in associations of risk factors with disease outcomes. Each center’s institutional review board approved the study, and all patients provided written informed consent. This study followed the Strengthening the Reporting of Observational Studies in Epidemiology (STROBE) guideline for reporting methods, findings, and study limitations for cohort studies.

Plasma levels of TMAO and related metabolites were measured in samples collected from 5418 participants at baseline (1989-1990 or 1992-1993) and again in samples collected from January 1996 to December 1997 for participants with a clinic visit at that time. Participants with prevalent diabetes (n = 887), missing diabetes status (n = 62), or antibiotic use (n = 18) at the time of their first TMAO level measurement were excluded from the study. Follow-up diabetes information was available in all but 9 participants, leaving 4442 participants in the analysis. Of those, 2226 participants had 2 measurements of metabolite levels. Participants were followed up to June 2010.

### Data Collection

Information on lifestyle and clinical risk factors was collected at baseline and annual study clinic visits.^28^ Medication use was assessed by a validated medication inventory.^29^ Plasma concentrations of glucose, insulin, lipids, and inflammatory biomarkers were assessed using enzymatic methods.^28^ Fasting glucose levels were measured in blood samples collected in the study years 1989 to 1990, 1992 to 1993, 1996 to 1997, 1998 to 1999, and 2005 to 2006; nonfasting plasma glucose levels were measured in 1994 to 1995. The Gutt ISI was calculated as *m*/(*G* × *I*), where *m* is a measure of glucose uptake during the oral glucose tolerance test calculated from body weight and from fasting and 2-hour glucose levels; *G* is the mean of fasting and 2-hour glucose levels; and *I* is a log_10_ transformation of the mean of fasting and 2-hour insulin levels.^27^ Hemoglobin A_1c_ levels were measured in a subset of 1094 CHS participants from the North Carolina enrollment site using blood samples collected in 1989 to 1990.^30^ Body mass index (BMI) was calculated from body weight in kilograms divided by height in meters squared. Estimated glomerular filtration rate (eGFR) was calculated with an equation from the Chronic Kidney Disease Epidemiology Collaboration using serum creatinine and cystatin C levels.^31^ Physical activity (kilocalories per week) was assessed using a modified Minnesota Leisure-Time Activities questionnaire.^32^ Dietary habits were assessed at baseline using a validated picture-sort food frequency questionnaire^33^ and in 1995 to 1996 using a validated 131-item food frequency questionnaire.^34^ Animal-sourced foods included processed meat, unprocessed red meat, chicken, fish, and eggs.

### Measurement of TMAO and Related Metabolites

The metabolites were measured in EDTA-plasma samples that were stored at −80°C. Trimethylamine *N*-oxide is stable during long-term storage at −80°C and several freeze/thaw cycles.^35^ Concentrations of TMAO, choline, carnitine, betaine, γ-butyrobetaine, and crotonobetaine were quantified by stable isotope dilution high-performance liquid chromatography with online tandem mass spectrometry as reported previously.^3^ The coefficients of variation of the laboratory assay across the study period varied over batches to a maximum of 5.8% for TMAO, 4.1% for choline, 5.7% for carnitine, 7.0% for betaine, 7.9% for γ-butyrobetaine, and 19.8% for crotonobetaine.

### Ascertainment of Diabetes

Diabetes was defined by a fasting (≥8 hours) plasma glucose level of at least 126 mg/dL or random (<8 hours fasting) glucose levels of at least 200 mg/dL (to convert to mmol/L, multiply by 0.0555) or new use of insulin or oral hypoglycemic medication (assessed annually). Starting at the 1992-1993 annual visit, diabetes was also identified using the Centers for Medicare & Medicaid Services records. Diabetes from the Centers for Medicare & Medicaid Services records was defined by at least 2 inpatient (ie, hospital, nursing home, or home health services), at least 3 outpatient (outpatient or carrier health services), or at least 1 inpatient and at least 1 outpatient *International Classification of Diseases, Ninth Revision*, claim codes for diabetes diagnosis (250.xx) during a 2-year period.^36^

### Measurements of Insulin Resistance and Sensitivity

We observed that fasting insulin level was highly correlated with homeostatic model assessment of insulin resistance (HOMA-IR) in the CHS cohort (ρ > 0.95), indicating that fasting insulin level was an estimate of insulin resistance. For this reason, we did not evaluate HOMA-IR separately. Fasting measures of insulin resistance generally reflect hepatic insulin sensitivity. We also used the Gutt ISI, which is a measure of post–glucose loading insulin resistance and reflects whole-body insulin sensitivity.^27^

### Statistical Analysis

Statistical analyses were conducted primarily from July 2019 to September 2020. Linear regression with robust SEs was used to investigate cross-sectional associations of quintiles of TMAO and related metabolites with log-transformed fasting insulin level, fasting glucose level, the Gutt ISI, and, in a subset with the available data, hemoglobin A_1c_ level. Quintiles were used to minimize the potential influence of outliers. For participants with sets of measurements taken at 2 times, both times were used and within-participant correlation was accounted for using generalized estimating equations with an independence working correlation matrix and robust SEs.^37^ We adjusted for covariates assessed at the time of each exposure measurement. We fit prespecified models for each metabolite. Model 1 (primary model) included adjustments for age, sex, race (White vs other), enrollment site, educational attainment (high school graduate, some college, and college graduate vs less than high school graduate), income ($12 000-$24 900, $25 000-$49 900, and >$50 000 vs <$12 000), BMI, waist circumference, smoking status (never, former, or current), physical activity (kilocalories per week of energy expenditure), systolic blood pressure, treated hypertension, low-density lipoprotein cholesterol level, prevalent coronary heart disease, daily number of servings of animal-sourced foods, and total energy intake (calories per day). Model 2 included further adjustment for eGFR.

For prospective investigation of incident diabetes, Cox proportional hazards regression was used for each metabolite to estimate the hazard ratios (HRs) for incident diabetes comparing each of the 4 higher metabolite level quintiles as indicator variables with the lowest (reference) quintile. The time variable in these analyses was time since the first TMAO measurement, with 361 participants missing metabolite measurements at baseline left entered in 1996 to 1997, the time of their first metabolite measurement. Participants remained at risk until the first diabetes diagnosis, death, or censoring at latest date of diabetes surveillance (2010) or loss to follow-up. Quintile cut points for each metabolite were based on the distribution in the cohort at study baseline. Hazard ratio estimates were based on indicator-variable models for the quintiles, but the hypothesis test for each metabolite was based on a linear trend model. We fit models as described for the linear regression analyses. For participants with metabolite measurements at 2 times, levels of metabolites and adjustment covariates were updated to the most recent value at the time of the second measurement. The prospective analyses did not provide evidence of departure from the proportional hazard assumption. We conducted additional analyses restricted to the median follow-up (12 years) to explore whether associations might be observed with shorter follow-up.

For each metabolite, we explored potential effect modification by age (in years), sex (binary), coronary heart disease (yes or no), evidence of kidney disease based on an eGFR of less than 60 mL/min (yes or no), BMI, and number of servings of animal-sourced food consumed in models that included a multiplicative term between the metabolite (linear variable) and the effect modifier. Linear variables were set at the median value for the effect modifier.

Missing covariate data (0.1% to 19.5% at baseline; 1.7% to 20.2% in 1996-1997) were imputed using multiple demographic and risk variables with single multivariable imputation, which prior analyses in the CHS have shown produces very similar results to those of multiple imputation.^38^ To correct for multiple comparisons, the significance level of main-effects tests was set as 2-tailed α = .0083 (.05/6 metabolites); and the significance level of interaction tests was set at α = .0014 (.05/36 [6 metabolites × 6 effect modifiers]). Analyses were performed using STATA, version 16.0 (StataCorp LLC).

## Results

Among the 4442 participants at baseline, the mean (SD) age was 73 (6) years, 2710 (61%) were women, 1732 (39%) were men, and 621 (14%) were Black. Characteristics of the CHS participants and mean plasma concentrations of the metabolites are provided in Table 1. Spearman correlation coefficients of TMAO with the other metabolites were 0.20 (choline), 0.05 (betaine), 0.18 (carnitine), 0.26 (γ-butyrobetaine), and 0.30 (crotonobetaine). Correlation coefficients between the metabolites other than TMAO ranged from 0.22 to 0.39 (eTable 1 in the Supplement).

**Table 1.** Characteristics and Metabolite Plasma Concentrations Among 4442 Participants in the Cardiovascular Health Study

Characteristic	Study participants^a^	
	Women (n = 2710)	Men (n = 1732)
Age, y	73 (6)	74 (6)
Race, No. (%)		
White	2292 (85)	1502 (87)
Black	402 (15)	219 (13)
Prevalent cardiovascular heart disease, No. (%)	396 (15)	430 (25)
Prevalent atrial fibrillation, No. (%)	58 (2)	98 (6)
Systolic blood pressure, mm Hg	136 (22)	134 (21)
Treated hypertension, No. (%)	1244 (46)	768 (44)
Past smoking, No. (%)	839 (31)	1001 (58)
Current smoking, No. (%)	340 (13)	197 (11)
BMI	26 (5)	26 (4)
Physical activity, kcal/wk	872 (1214)	1619 (1850)
Glucose level, mg/dL	98 (10)	101 (10)
Insulin level, μIU/mL	14 (11)	14 (7)
Hemoglobin A_1c_ level, %^b^	6.1 (1.0)	6.1 (1.3)
Gutt ISI^c^	60 (23)	63 (25)
LDL cholesterol level, mg/dL	134 (35)	125 (33)
eGFR, mL/min	80 (20)	74 (18)
Animal-sourced food intake, servings/d^d^	1.6 (0.9)	1.8 (1.0)
Fruit intake, servings/d	2.3 (1.1)	2.0 (1.0)
Vegetable intake, servings/d	2.7 (1.4)	2.4 (1.4)
Plasma concentration, μmol/L		
TMAO	6.9 (9.2)	8.2 (12.1)
Choline	9.5 (3.4)	10.7 (3.8)
Betaine	35.0 (12.0)	43.0 (13.0)
Carnitine	37.0 (8.0)	39.0 (8.0)
γ-Butyrobetaine	0.9 (0.3)	1.2 (0.4)
Crotonobetaine	0.02 (0.02)	0.03 (0.02)

Abbreviations: BMI, body mass index (calculated as weight in kilograms divided by height in meters squared); eGFR, estimated glomerular filtration rate; ISI, insulin sensitivity index; LDL, low-density lipoproteins; TMAO, trimethylamine *N*-oxide.

SI conversion factors: To convert LDL cholesterol to mmol/L, multiply by 0.0259; glucose to mmol/L, multiply by 0.0555; hemoglobin A_1c_ to proportion of total hemoglobin, multiply by 0.01; and insulin to pmol/L, multiply by 6.945.

^a^ Unless otherwise indicated, data are expressed as mean (SD).

^b^ Available in 522 women and 312 men.

^c^ Available in 2293 women and 1529 men.

^d^ Includes meat, chicken, fish, and eggs.

In cross-sectional analyses, high concentrations of plasma TMAO were associated with high fasting insulin levels (Table 2). Compared with the first quintile, participants in the fifth quintile had 7% higher mean insulin levels (95% CI, 4%-10%; *P* < .001) after multivariable adjustments (geometric mean ratio, 1.07; 95% CI, 1.04-1.10). Adjustment for eGFR, a marker of kidney function and potential confounder or mediator of TMAO,^39,40,41^ greatly reduced the TMAO-insulin association. Trimethylamine *N*-oxide was not significantly associated with plasma glucose levels nor the Gutt ISI at the prespecified *P* value threshold of .008 (Table 2).

**Table 2.** Association of Serial Measures of Plasma TMAO and Related Metabolites With Plasma Fasting Insulin and Glucose Levels and the Gutt Insulin Sensitivity Index Among 4428 Participants in the Cardiovascular Health Study

Metabolite	Quintile^a^					*P* value for trend
	1	2	3	4	5	
**TMAO**						
Concentration range, μmol/L	0.01 to 2.94	2.94 to 4.07	4.01 to 5.60	5.60 to 8.76	8.78 to 235.63	NA
Plasma fasting insulin level, GMR (95% CI)^b^						
Model 1	1 [Reference]	1.02 (0.99 to 1.05)	1.05 (1.02 to 1.08)	1.08 (1.05 to 1.11)	1.07 (1.04 to 1.10)	<.001
Model 2	1 [Reference]	1.01 (0.98 to 1.04)	1.03 (1.00 to 1.06)	1.05 (1.02 to 1.08)	1.02 (0.99 to 1.05)	.02
Plasma fasting glucose level, mg/dL, mean difference (95% CI)^c^						
Model 1	0 [Reference]	0.57 (−0.16 to 1.30)	0.42 (−0.28 to 1.13)	0.93 (0.18 to 1.68)	0.71 (−0.04 to 1.46)	.04
Model 2	0 [Reference]	0.62 (−0.12 to 1.35)	0.52 (−0.20 to 1.23)	1.07 (0.30 to 1.84)	0.91 (0.13 to 1.69)	.01
Plasma Gutt ISI, mean difference (95% CI)^d^						
Model 1	0 [Reference]	−0.47 (−2.46 to 1.51)	−0.37 (−2.25 to 1.51)	−0.14 (−2.13 to 1.84)	0.17 (−1.86 to 2.20)	.78
Model 2	0 [Reference]	−0.37 (−2.36 to 1.62)	−0.13 (−2.04 to 1.77)	0.22 (−1.80 to 2.23)	0.67 (−1.46 to 2.79)	.44
**Choline**						
Concentration range, μmol/L	0.24 to 7.76	7.76 to 8.90	8.90 to 10.06	10.06 to 11.59	11.59 to 111.06	NA
Plasma fasting insulin level, GMR (95% CI)^b^						
Model 1	1 [Reference]	0.99 (0.96 to 1.02)	0.98 (0.96 to 1.01)	0.99 (0.96 to 1.02)	1.00 (0.97 to 1.03)	.95
Model 2	1 [Reference]	0.98 (0.95 to 1.01)	0.97 (0.94 to 1.00)	0.96 (0.93 to 0.99)	0.95 (0.92 to 0.98)	<.001
Plasma fasting glucose level, mg/dL, mean difference (95% CI)^c^						
Model 1	0 [Reference]	−0.35 (−1.11 to 0.40)	−0.62 (−1.37 to 0.13)	−1.13 (−1.92 to −0.34)	−1.22 (−1.99 to −0.45)	<.001
Model 2	0 [Reference]	−0.36 (−1.12 to 0.40)	−0.62 (−1.38 to 0.13)	−1.13 (−1.94 to −0.33)	−1.23 (−2.05 to −0.41)	.001
Plasma Gutt ISI, mean difference (95% CI)^d^						
Model 1	0 [Reference]	1.42 (−0.58 to 3.41)	0.51 (−1.50 to 2.53)	2.60 (0.53 to 4.68)	2.27 (0.16 to 4.38)	.02
Model 2	0 [Reference]	1.57 (−0.43 to 3.57)	0.74 (−1.30 to 2.77)	2.97 (0.87 to 5.08)	2.96 (0.75 to 5.18)	<.001
**Betaine**						
Concentration range, μmol/L	0.41 to 27.43	27.43 to 33.52	33.53 to 39.20	39.21 to 46.91	46.91 to 167.64	NA
Plasma fasting insulin level, GMR (95% CI)^b^						
Model 1	1 [Reference]	1.00 (0.97 to 1.03)	0.98 (0.96 to 1.01)	0.94 (0.91 to 0.97)	0.93 (0.90 to 0.96)	<.001
Model 2	1 [Reference]	1.00 (0.97 to 1.03)	0.98 (0.95 to 1.01)	0.94 (0.91 to 0.96)	0.93 (0.90 to 0.96)	<.001
Plasma fasting glucose level, mg/dL, mean difference (95% CI)^c^						
Model 1	0 [Reference]	0.41 (−0.33 to 1.15)	0.44 (−0.31 to 1.19)	−0.22 (−0.97 to 0.54)	−0.29 (−1.08 to 0.51)	.19
Model 2	0 [Reference]	0.41 (−0.33 to 1.15)	0.45 (−0.30 to 1.20)	−0.21 (−0.96 to 0.55)	−0.28 (−1.07 to 0.52)	.20
Plasma Gutt ISI, mean difference (95% CI)^d^						
Model 1	0 [Reference]	2.50 (0.61 to 4.40)	2.40 (0.49 to 4.30)	4.16 (2.11 to 6.22)	6.46 (4.32 to 8.60)	<.001
Model 2	0 [Reference]	2.50 (0.61 to 4.40)	2.45 (0.55 to 4.36)	4.23 (2.17 to 6.28)	6.53 (4.39 to 8.67)	<.001
**Carnitine**						
Concentration range, μmol/L	1.22 to 31.08	31.10 to 35.13	35.13 to 39.04	39.06 to 44.05	44.08 to 94.99	NA
Plasma fasting insulin level, GMR (95% CI)^b^						
Model 1	1 [Reference]	1.01 (0.99 to 1.04)	1.04 (1.01 to 1.07)	1.05 (1.02 to 1.08)	1.07 (1.03 to 1.10)	<.001
Model 2	1 [Reference]	1.01 (0.99 to 1.04)	1.03 (1.00 to 1.06)	1.04 (1.01 to 1.07)	1.04 (1.01 to 1.08)	<.001
Plasma fasting glucose level, mg/dL, mean difference (95% CI)^c^						
Model 1	0 [Reference]	1.03 (0.33 to 1.73)	1.24 (0.53 to 1.95)	1.35 (0.61 to 2.10)	1.95 (1.20 to 2.70)	<.001
Model 2	0 [Reference]	1.04 (0.34 to 1.73)	1.27 (0.56 to 1.98)	1.40 (0.66 to 2.14)	2.04 (1.28 to 2.80)	<.001
Plasma Gutt ISI, mean difference (95% CI)^d^						
Model 1	0 [Reference]	−1.01 (−2.92 to 0.90)	0.13 (−1.79 to 2.06)	0.54 (−1.48 to 2.56)	−0.05 (−2.10 to 2.01)	.55
Model 2	0 [Reference]	−0.99 (−2.90 to 0.91)	0.19 (−1.73 to 2.12)	0.65 (−1.37 to 2.66)	0.16 (−1.91 to 2.23)	.43
**γ-Butyrobetaine**						
Concentration range, μmol/L	0.01 to 0.77	0.77 to 0.92	0.92 to 1.07	1.07 to 1.28	1.28 to 4.61	NA
Plasma fasting insulin level, GMR (95% CI)^b^						
Model 1	1 [Reference]	1.04 (1.01 to 1.06)	1.02 (0.99 to 1.05)	1.04 (1.01 to 1.07)	1.06 (1.02 to 1.09)	.001
Model 2	1 [Reference]	1.02 (0.99 to 1.05)	0.99 (0.96 to 1.02)	1.00 (0.97 to 1.03)	0.99 (0.96 to 1.02)	.28
Plasma fasting glucose level, mg/dL, mean difference (95% CI)^c^						
Model 1	0 [Reference]	0.30 (−0.42 to 1.02)	−0.04 (−0.78 to 0.71)	0.50 (−0.28 to 1.29)	−0.47 (−1.30 to 0.36)	.53
Model 2	0 [Reference]	0.33 (−0.39 to 1.06)	0.02 (−0.75 to 0.78)	0.59 (−0.23 to 1.40)	−0.34 (−1.24 to 0.56)	.80
Plasma Gutt ISI, mean difference (95%)^d^						
Model 1	0 [Reference]	0.40 (−1.50 to 2.30)	2.68 (0.64 to 4.71)	2.27 (0.15 to 4.39)	2.70 (0.41 to 4.98)	.01
Model 2	0 [Reference]	0.72 (−1.19 to 2.63)	3.22 (1.16 to 5.28)	3.09 (0.94 to 5.24)	3.95 (1.55 to 6.36)	<.001
**Crotonobetaine**						
Concentration range, μmol/L	0.008 to 0.010	0.020 to 0.021	0.022 to 0.026	0.027 to 0.033	0.034 to 0.314	NA
Plasma fasting insulin level, GMR (95% CI)^b^						
Model 1	1 [Reference]	1.00 (0.97 to 1.03)	1.01 (0.98 to 1.03)	1.04 (1.02 to 1.07)	1.05 (1.02 to 1.08)	<.001
Model 2	1 [Reference]	0.99 (0.96 to 1.02)	0.99 (0.96 to 1.01)	1.01 (0.98 to 1.04)	1.00 (0.97 to 1.03)	.92
Plasma fasting glucose level, mg/dL, mean difference (95% CI)^c^						
Model 1	0 [Reference]	0.13 (−0.67 to 0.92)	0.25 (−0.38 to 0.87)	0.38 (−0.31 to 1.07)	0.58 (−0.16 to 1.33)	.10
Model 2	0 [Reference]	0.18 (−0.61 to 0.98)	0.34 (−0.30 to 0.97)	0.54 (−0.17 to 1.25)	0.84 (0.04 to 1.64)	.03
Plasma Gutt ISI, mean difference (95% CI)^d^						
Model 1	0 [Reference]	0.47 (−1.71 to 2.65)	1.60 (−0.08 to 3.29)	0.59 (−1.28 to 2.46)	−0.31 (−2.27 to 1.65)	.84
Model 2	0 [Reference]	0.59 (−1.59 to 2.77)	1.81 (0.12 to 3.49)	0.95 (−0.98 to 2.87)	0.26 (−1.86 to 2.38)	.43

Abbreviations: GMR, geometric mean ratio; ISI, insulin sensitivity index; NA, not applicable; TMAO, trimethylamine *N*-oxide.

SI conversion factors: To convert glucose to mmol/L, multiply by 0.0555.

^a^ Model 1 adjusted for age, sex, race, site, educational attainment, income, body mass index, waist circumference, smoking, physical activity, systolic blood pressure, hypertension, low-density lipoprotein cholesterol level, coronary heart disease, animal-sourced food consumption, and total energy intake. Model 2 additionally adjusted for estimated glomerular filtration rate.

^b^ Compares each of the 4 higher metabolite quintiles with the lowest (reference) quintile. The GMR was obtained in cross-sectional analyses, using generalized estimating equations in models with serial insulin, metabolite, and covariate measurements.

^c^ Indicates mean difference in plasma glucose level comparing each of the 4 higher metabolite quintiles with the lowest (reference) quintile. The mean differences were obtained in cross-sectional analyses using generalized estimating equations in models with serial glucose, metabolite, and covariate measurements.

^d^ Indicates mean difference in plasma Gutt ISI comparing each of the 4 higher metabolite quintiles with the lowest (reference) quintile. The mean differences were obtained in cross-sectional analyses using generalized estimating equations in models with serial Gutt ISI, metabolite, and covariate measurements.

High concentrations of plasma carnitine (geometric mean ratio for highest vs lowest quintiles, 1.07; 95% CI, 1.03-1.10), γ-butyrobetaine (geometric mean ratio for highest vs lowest quintiles, 1.06; 95% CI, 1.02-1.09), and crotonobetaine (geometric mean ratio for highest quintile, 1.05; 95% CI, 1.02-1.08) were each associated with increased fasting insulin levels (Table 2). After further adjustment for eGFR, the associations for γ-butyrobetaine and crotonobetaine were no longer statistically significant, whereas the association of carnitine remained significant (geometric mean ratio for highest vs lowest quintiles, 1.04; 95% CI, 1.01-1.08). High concentration of carnitine was also associated with increased glucose concentrations independent of eGFR adjustment (mean difference for highest vs lowest quintiles, 1.95; 95% CI, 1.20-2.70) (Table 2). In contrast, betaine and choline were associated with decreased fasting insulin levels in models adjusted for eGFR (geometric mean ratio for highest vs lowest quintiles, 0.93 [95% CI, 0.90-0.96] and 0.95 [95% CI, 0.92-0.98], respectively), decreased glucose levels for choline only (mean difference for highest vs lowest quintiles, −1.22; 95% CI, −1.99 to −0.45), and increased Gutt ISI (mean difference for highest vs lowest quintiles, 6.46 [95% CI, 4.32-8.60] and 2.27 [95% CI, 0.16-4.38], respectively) (Table 2). High concentration of γ-butyrobetaine was also associated with increased Gutt ISI (mean difference for highest vs lowest quintiles, 2.70; 95% CI, 0.41-4.98]) (Table 2). A summary of these findings in the context of their general metabolite interrelationships is shown in the Figure.

**Figure.**
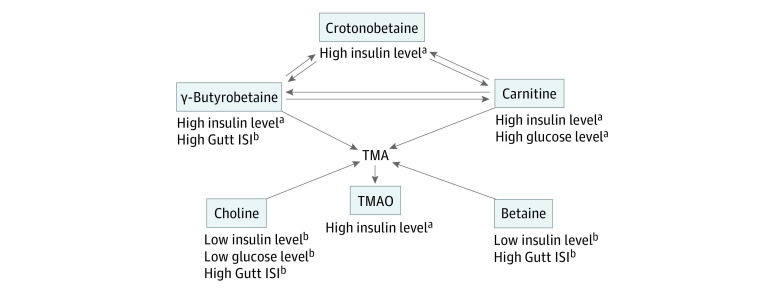
Associations of Metabolites With Plasma Fasting Insulin and Glucose Levels and the Estimated Gutt Insulin Sensitivity Index (ISI) Population includes 4442 participants in the Cardiovascular Health Study. The arrows represent simplified pathways of the 6 metabolites that were measured in the study, shown in boxes. The gut microbiota generates trimethylamine (TMA) from dietary carnitine, betaine, or choline, and the absorbed TMA is transformed into trimethylamine *N*-oxide (TMAO) by the liver flavin monooxygenase 3. γ-Butyrobetaine and crotonobetaine are 2 other metabolites of carnitine produced by the gut microbiota. The statistically significant associations of higher concentrations of the metabolites with plasma fasting levels of insulin and glucose and the Gutt ISI were observed in multivariable models and detailed in Table 2 and Table 3. ^a^Outcome expected to worsen glucose-insulin homeostasis. ^b^Outcome expected to improve glucose-insulin homeostasis.

In a subset of 769 CHS participants, we did not observe significant associations of any of the metabolites with levels of hemoglobin A_1c_ (eTable 2 in the Supplement). In exploratory analyses, we observed a significant modification of the association of betaine with glucose by sex (*P* < .001 for interaction): a high concentration of plasma betaine was significantly associated with decreased fasting glucose level in men but not women. Comparing the highest quintile with the lowest, the mean difference was 1.85 (95% CI, −3.36 to −0.33) and 0.07 (95% CI. −0.98 to 1.11) in men and women, respectively (eTable 3 in the Supplement).

We identified 661 cases of incident diabetes during follow-up (median, 12.0 [range, 6.9-17.1] years) for an incidence rate of 12.5 per 1000 person-years. Plasma concentrations of TMAO were not associated with incident diabetes (Table 3). After multivariable adjustments, the HR of diabetes associated with TMAO concentrations in the fifth quintile compared with the first quintile was 1.20 (95% CI, 0.94-1.55; *P* = .46). Further adjustment for eGFR did not materially change these results (HR, 1.19; 95% CI, 0.92-1.55) (Table 3). Plasma concentrations of carnitine (HR, 1.07; 95% CI, 0.83-1.37), betaine (HR, 0.88; 95% CI, 0.67-1.15), choline (HR, 0.96; 95% CI, 0.74-1.24), γ-butyrobetaine (HR, 0.79; 95% CI, 0.60-1.04), and crotonobetaine (HR, 1.06; 95% CI, 0.83-1.35) were also not significantly associated with incident diabetes (Table 3). Findings were similar in exploratory analyses, with follow-up restricted to the first 12 years (eTable 4 in the Supplement). The association between the metabolites and incident diabetes was not significantly different by age, sex, BMI, coronary heart disease, eGFR, or dietary intake of animal-sourced foods (eTable 5 in the Supplement).

**Table 3.** Association of Serial Measurements of Plasma TMAO and Related Metabolites With Incident Type 2 Diabetes Among 4442 Participants in the Cardiovascular Health Study

Metabolite	Quintile, HR (95% CI)^a^					*P* value for trend
	1	2	3	4	5	
TMAO						
Model 1	1 [Reference]	1.11 (0.86-1.42)	1.21 (0.95-1.54)	0.95 (0.74-1.23)	1.20 (0.94-1.55)	.46
Model 2	1 [Reference]	1.10 (0.86-1.42)	1.21 (0.95-1.53)	0.95 (0.73-1.23)	1.19 (0.92-1.55)	.52
Choline						
Model 1	1 [Reference]	1.08 (0.83-1.40)	1.00 (0.77-1.30)	0.95 (0.73-1.22)	0.96 (0.74-1.24)	.45
Model 2	1 [Reference]	1.07 (0.83-1.39)	0.99 (0.77-1.29)	0.93 (0.72-1.21)	0.94 (0.72-1.22)	.36
Betaine						
Model 1	1 [Reference]	1.17 (0.93-1.48)	1.03 (0.81-1.32)	0.88 (0.68-1.14)	0.88 (0.67-1.15)	.07
Model 2	1 [Reference]	1.17 (0.93-1.49)	1.03 (0.80-1.31)	0.88 (0.68-1.14)	0.88 (0.67-1.15)	.07
Carnitine						
Model 1	1 [Reference]	1.17 (0.92-1.50)	1.08 (0.85-1.38)	1.16 (0.91-1.48)	1.07 (0.83-1.37)	.64
Model 2	1 [Reference]	1.17 (0.91-1.50)	1.08 (0.85-1.38)	1.16 (0.91-1.48)	1.06 (0.82-1.37)	.68
γ-Butyrobetaine						
Model 1	1 [Reference]	0.80 (0.63-1.01)	0.72 (0.57-0.92)	0.82 (0.64-1.05)	0.79 (0.60-1.04)	.13
Model 2	1 [Reference]	0.79 (0.62-1.00)	0.70 (0.55-0.90)	0.79 (0.61-1.02)	0.74 (0.55-1.00)	.07
Crotonobetaine						
Model 1	1 [Reference]	1.04 (0.79-1.37)	1.05 (0.85-1.30)	1.12 (0.89-1.41)	1.06 (0.83-1.35)	.45
Model 2	1 [Reference]	1.04 (0.79-1.37)	1.05 (0.85-1.30)	1.11 (0.88-1.40)	1.05 (0.82-1.35)	.50

Abbreviations: HR, hazard ratio; TMAO, trimethylamine *N*-oxide.

^a^ Hazard ratios for incident type 2 diabetes comparing each of the 4 higher metabolite quintiles with the lowest (reference) quintile, obtained from Cox proportional hazards regression models with serial measures of metabolite and time-updated covariates. Model 1 adjusted for age, sex, race, site, educational attainment, income, body mass index, waist circumference, smoking, physical activity, systolic blood pressure, hypertension, low-density lipoprotein cholesterol, coronary heart disease, animal-sourced food consumption, and total energy intake. Model 2 additionally adjusted for estimated glomerular filtration rate.

## Discussion

In this large, prospective study among older adults, we evaluated how plasma concentrations of TMAO, carnitine, choline, betaine, γ-butyrobetaine, and crotonobetaine were associated with the risk of incident diabetes in a community-based cohort. We observed no significant associations of the metabolites with risk of incident diabetes. When we evaluated markers of glucose-insulin homeostasis as potentially more sensitive early measures of metabolic dysfunction, several cross-sectional associations with markers of glucose-insulin homeostasis were identified.

### Trimethylamine *N*-Oxide

Evidence of an association of TMAO with diabetes in humans has been inconsistent. Trimethylamine *N*-oxide is associated with prevalent diabetes in cross-sectional analyses,^21,22,42^ and in a hospital-based retrospective case-control study,^23^ elevated plasma TMAO levels were associated with an increased risk of diabetes. However, in a case-cohort study nested in the Prevención con Dieta Mediterránea (PREDIMED) trial, high plasma TMAO concentrations were associated with a decreased risk of incident diabetes.^26^ In contrast, in a prospective study among Norwegian patients with stable angina, plasma TMAO levels were not significantly associated with incident diabetes after adjustment for other risk factors.^25^ Our study builds on and extends these prior findings in several important respects. First, compared with prior retrospective studies, our design minimizes reverse causation and selection bias. Second, each of these prior studies only used a single TMAO measure at baseline. In our study, the within-person Spearman correlation coefficient for repeated TMAO measures over up to 7 years of follow-up was 0.25; corresponding correlation coefficients for the other metabolites ranged from 0.33 to 0.59. These results suggest significant intraperson variability in diet, microbiome, and/or metabolism over many years, so that a single baseline measure of these metabolites may not accurately reflect long-term exposure (or corresponding clinical risk). Thus, compared with prior studies, our use of serial measures strengthens the precision of our exposure measurement and validity of findings. Third, we carefully adjusted for many covariates reducing the effect of residual confounding, and we evaluated the effect of adjusting for eGFR. Fourth, we evaluated not only TMAO but also 5 closely related precursors and intermediates, providing a more complete picture of this family of diet- and microbiome-related molecules. Finally, the CHS is a general, community-based, prospective cohort of older adults, greatly increasing generalizability compared with some of the prior selected populations. Our findings and those of prior studies do not support the hypothesis that TMAO or its related metabolites increase the risk of incident diabetes.

Although not associated with incident diabetes, TMAO showed a cross-sectional positive association with fasting insulin level, a marker of insulin resistance. This finding is consistent with animal data^19,20^ and with reported associations of TMAO with HOMA-IR in the Multiethnic Cohort Adiposity Phenotype Study.^43^ In the Oral Infections, Glucose Intolerance and Insulin Resistance Study (ORIGINS), plasma TMAO level was not associated with HOMA-IR.^44^ Participants in ORIGINS were young adults (mean [SD] age, 34 [10] years), with TMAO concentrations only 57% as high as in the CHS, and associations may differ in young adults, who are much less likely to be in the early stages of developing diabetes. Of note, the association of TMAO with insulin in CHS was greatly diminished by adjustment for eGFR. As observed in other studies,^40^ circulating levels of TMAO were elevated in CHS participants with low eGFR, raising the possibility that kidney function may confound the association of TMAO with insulin. However, an elevated TMAO level itself leads to an elevated cystatin C level, reduced renal filtration, and ultimately renal dysfunction in mice; conversely, a reduction in TMAO levels attenuates renal functional decline in animal models of chronic kidney disease.^39,40^ Therefore, heightened TMAO levels may have a direct adverse effect on renal function, and eGFR could also be a mediator of the TMAO-insulin association. The CHS results cannot distinguish between these possibilities.

### Other Metabolites

The metabolites we measured are related at least in part to TMAO (Figure). Choline, betaine, and carnitine are dietary precursors of TMAO. The gut microbiota generates TMA from these precursors and the absorbed TMA is transformed into TMAO by the host liver. Carnitine is also a precursor of crotonobetaine and γ-butyrobetaine, both produced by the gut microbiota. Interestingly, plasma levels of choline and betaine were each associated with better glucose homeostasis markers, whereas carnitine and its 3 microbiota-generated metabolites were each associated with increased fasting insulin levels (summarized in the Figure). Although the associations of carnitine persisted with adjustment for eGFR, the associations of TMAO, crotonobetaine, and γ-butyrobetaine were each attenuated by eGFR adjustment, suggesting either confounding or mediation by kidney function. These novel findings suggest a need for mechanistic studies to evaluate the effects of carnitine and its microbiome-generated metabolites on insulin resistance, as well as the interplay of renal function.

High plasma concentrations of choline were associated with decreased fasting glucose and insulin levels and increased insulin sensitivity, whereas high plasma concentrations of betaine were associated with decreased fasting insulin levels and increased insulin sensitivity. Although dietary supplementation with choline markedly increases plasma TMAO levels in mice^45^ and in humans,^46^ circulating choline was only modestly correlated with plasma TMAO concentration (ρ = 0.20 in CHS). Likewise, circulating betaine was not correlated with TMAO concentration (ρ = 0.05), suggesting the associations of plasma choline and plasma betaine may have been independent of their precursor-product relationship with TMAO. Choline also plays essential biological roles, including incorporation into membrane phosphatidylcholine and sphingomyelin, and it is a precursor of betaine for the 1-carbon pathway. Similar associations for choline as for betaine may reflect their precursor-product relationship in this pathway.^47^

The lack of significant association of betaine with incident diabetes in the CHS contrasts with findings of prospective studies in selected populations.^25,26,48^ In particular, elevated plasma betaine level was associated with a decreased risk of incident diabetes in the study of Norwegian patients with stable angina,^25^ in the PREDIMED trial,^26^ and in the Prevention of Renal and Vascular End-Stage Disease (PREVEND) study.^48^ Differences in populations, including age, lifestyle, and selection criteria, may contribute to discrepancies in results. In the PREVEND study, the association of betaine with a lower risk of diabetes was observed only in men. We did not find a significant betaine-sex interaction for diabetes risk in the CHS, but we observed a betaine-sex interaction for plasma glucose level: the association of betaine with lower plasma glucose level was observed only in men. A high concentration of plasma betaine was also associated with a decreased fasting insulin level and increased index of insulin sensitivity. In light of prior results, our study suggests that betaine, or other factors in its food sources of shellfish, wheat germ and bran, and spinach, could have protective effects for insulin resistance and insulin sensitivity, highlighting the need for further investigation.

### Limitations

This study has potential limitations. The CHS population consists of older adults, and findings may not generalize to younger populations. Adiposity measures and lifestyle factors such as physical (in)activity are associated with incident diabetes in the CHS,^49,50^ suggesting insulin resistance plays a role in the pathophysiology of type 2 diabetes in this population, as it does in younger populations. On the other hand, evidence of heterogeneity in the diabetes phenotype^51,52^ suggests impaired insulin secretion may be a contributing factor to some incident diabetes events in CHS. Older adults present an important, growing, and generally understudied subgroup at particular risk for poor metabolic health, and whether the pathophysiology of diabetes differs in older adults warrants further study.

Because this was an observational study, residual confounding could not be excluded. However, we adjusted for major known diabetes risk factors to reduce the effect of confounding. Variability over time in the metabolite measurement limited the precision of the estimated chronic exposure to the metabolites. The findings for glucose, insulin, and insulin sensitivity were cross-sectional, preventing assessment of temporality. In addition, the associations with glucose homeostasis markers did not translate into associations with incident diabetes. Likely changes in levels of the metabolites during the long follow-up may have reduced our ability to detect associations with diabetes. However, repeated measures would have reduced such measurement error.

## Conclusions

In this cohort study among older adults, levels of plasma TMAO, carnitine, choline, betaine, γ-butyrobetaine, and crotonobetaine were not associated with incident diabetes. Cross-sectional findings suggest the possibility of early involvement of some of the metabolites with glucose-insulin homeostasis, which is detrimental for TMAO and carnitine and favorable for betaine and choline.

## Notes

Supplement. **eTable 1.** Spearman Correlation Coefficients Between Baseline Metabolite Plasma Concentrations Among 4442 Participants in the Cardiovascular Health Study **eTable 2.** Association of Serial Measures of Plasma TMAO and Related Metabolites With Plasma Hemoglobin A_1c_ Among 769 Participants in the Cardiovascular Health Study **eTable 3.** Association of Serial Measures of Plasma Betaine With Fasting Plasma Glucose Among 4442 Participants in the Cardiovascular Health Study, Stratified by Sex **eTable 4.** Association of Serial Measures of Plasma TMAO and Related Metabolites With Incident Type 2 Diabetes Among 4442 Participants in the Cardiovascular Health Study With Follow-up Restricted to the First 12 Years **eTable 5.** Tests of Interaction Between the Metabolites and Age, Sex, BMI, Coronary Heart Disease, eGFR, and Dietary Intake of Animal-Sourced Foods in the Analysis of Incident Type 2 Diabetes
