# Supplementary material for: Association of Trimethylamine N-Oxide and Related Metabolites in Plasma and Incident Type 2 Diabetes: The Cardiovascular Health Study
Source: JAMA Netw Open. 2021 Aug 27;4(8):e2122844. doi: 10.1001/jamanetworkopen.2021.22844 (PMC8397925; doi:10.1001/jamanetworkopen.2021.22844)
Supplement: Supplement. — eTable 1. Spearman Correlation Coefficients Between Baseline Metabolite Plasma Concentrations Among 4442 Participants in the Cardiovascular Health Study eTable 2. Association of Serial Measures of Plasma TMAO and Related Metabolites With Plasma Hemoglobin A1c Among 769 Participants in the Cardiovascular Health Study eTable 3. Association of Serial Measures of Plasma Betaine With Fasting Plasma Glucose Among 4442 Participants in the Cardiovascular Health Study, Stratified by Sex eTable 4. Association of Serial Measures of Plasma TMAO and Related Metabolites With Incident Type 2 Diabetes Among 4442 Participants in the Cardiovascular Health Study With Follow-up Restricted to the First 12 Years eTable 5. Tests of Interaction Between the Metabolites and Age, Sex, BMI, Coronary Heart Disease, eGFR, and Dietary Intake of Animal-Sourced Foods in the Analysis of Incident Type 2 Diabetes [file jamanetwopen-e2122844-s001.pdf]

Supplementary Online Content

Lemaitre RN, Jensen PN, Wang Z, et al. Association of trimethylamine *N*-oxide and related metabolites in plasma and incident type 2 diabetes: the Cardiovascular Health Study. *JAMA Netw Open*. 2021;4(8):e2122844.
doi:10.1001/jamanetworkopen.2021.22844

eTable 1. Spearman Correlation Coefficients Between Baseline Metabolite Plasma Concentrations Among 4442 Participants in the Cardiovascular Health Study
eTable 2. Association of Serial Measures of Plasma TMAO and Related Metabolites With Plasma Hemoglobin A_{1c} Among 769 Participants in the Cardiovascular Health Study
eTable 3. Association of Serial Measures of Plasma Betaine With Fasting Plasma Glucose Among 4442 Participants in the Cardiovascular Health Study, Stratified by Sex
eTable 4. Association of Serial Measures of Plasma TMAO and Related Metabolites With Incident Type 2 Diabetes Among 4442 Participants in the Cardiovascular Health Study With Follow-up Restricted to the First 12 Years
eTable 5. Tests of Interaction Between the Metabolites and Age, Sex, BMI, Coronary Heart Disease, eGFR, and Dietary Intake of Animal-Sourced Foods in the Analysis of Incident Type 2 Diabetes

This supplementary material has been provided by the authors to give readers additional information about their work.

© 2021 Lemaitre RN et al. *JAMA Network Open*.

eTable 1. Spearman Correlation Coefficients Between Baseline Metabolite Plasma Concentrations Among 4442 Participants in the Cardiovascular Health Study

	TMAO	Choline	Betaine	Carnitine	γ-Butyrobetaine
Choline	0.20	1			
Betaine	0.05	0.37	1		
Carnitine	0.18	0.26	0.27	1	
γ-Butyrobetaine	0.26	0.32	0.39	0.39	1
Crotonobetaine	0.30	0.32	0.22	0.27	0.33

Abbreviations: TMAO = Trimethylamine-N-oxide

© 2021 Lemaitre RN et al. *JAMA Network Open*.

eTable 2. Association of Serial Measures of Plasma TMAO and Related Metabolites With Plasma Hemoglobin A_{1c} Among 769 Participants in the Cardiovascular Health Study

	N	Mean difference[1]	95% CI	*P* value for trend
TMAO				
Q1	162	0	ref	0.05
Q2	172	-0.13	(-0.31, 0.04)	
Q3	158	0.06	(-0.16, 0.27)	
Q4	156	0.01	(-0.22, 0.24)	
Q5	121	0.22	(-0.04, 0.48)	
Choline				
Q1	110	0	ref	0.30
Q2	152	0.05	(-0.20, 0.30)	
Q3	167	-0.14	(-0.35, 0.07)	
Q4	170	-0.01	(-0.23, 0.20)	
Q5	170	0.17	(-0.08, 0.42)	
Betaine				
Q1	147	0	ref	0.24
Q2	146	-0.05	(-0.28, 0.17)	
Q3	164	-0.07	(-0.30, 0.17)	
Q4	144	-0.03	(-0.26, 0.20)	
Q5	168	-0.18	(-0.44, 0.09)	
Carnitine				
Q1	153	0	ref	0.49
Q2	147	0.09	(-0.16, 0.34)	
Q3	149	0.02	(-0.21, 0.25)	
Q4	168	0.23	(-0.00, 0.46)	
Q5	152	0.02	(-0.21, 0.25)	
Butyrobetaine				
Q1	152	0	ref	0.19
Q2	157	-0.08	(-0.27, 0.12)	
Q3	163	0.06	(-0.19, 0.32)	
Q4	144	-0.13	(-0.39, 0.12)	
Q5	153	-0.17	(-0.40, 0.07)	
Crotonobetaine				
Q1	259	0	ref	0.82
Q2	175	-0.12	(-0.33, 0.10)	
Q3	176	-0.05	(-0.23, 0.14)	
Q4	147	-0.01	(-0.27, 0.25)	

© 2021 Lemaitre RN et al. *JAMA Network Open*.

Q5	112	0.03	(-0.21, 0.28)	

[1] Mean difference in plasma hemoglobin A1c comparing each of the four higher metabolite quintiles to the lowest (reference) quintile. The mean differences were obtained in cross-sectional analyses using Generalized Estimating Equations in models with serial metabolite and covariate measurements. The multivariable model adjusted for age, sex, race, site, education, income, BMI, waist, smoking, physical activity, systolic blood pressure, hypertension, LDL, CHD, animal sourced foods consumption, total energy intake. Abbreviations: TMAO = Trimethylamine-N-oxide.

© 2021 Lemaitre RN et al. *JAMA Network Open*.

eTable 3. Association of Serial Measures of Plasma Betaine With Fasting Plasma Glucose Among 4442 Participants in the Cardiovascular Health Study, Stratified by Sex

	Women (N=2710)			Men (N=1732)		
	Mean difference[1]	95% CI	*P* value for trend	Mean difference	95% CI	*P* value for trend
Q1	0	- -	0.56	0	- -	0.003
Q2	0.41	(-0.43, 1.24)		-0.66	(-2.29, 0.98)	
Q3	0.72	(-0.16, 1.61)		-1.30	(-2.85, 0.26)	
Q4	0.50	(-0.41, 1.40)		-2.35	(-3.89, -0.81)	
Q5	0.07	(-0.98, 1.11)		-1.85	(-3.36, -0.33)	

[1] Mean difference in plasma glucose comparing each of the four higher metabolite quintiles to the lowest (reference) quintile. The mean differences were obtained in cross-sectional analyses using Generalized Estimating Equations in models with serial glucose, metabolite, and covariate measurements. The models were adjusted for age, sex, race, site, education, income, BMI, waist, smoking, physical activity, systolic blood pressure, hypertension, LDL, CHD, animal sourced foods consumption, total energy intake and eGFR, and stratified on sex.

Abbreviations: TMAO = Trimethylamine-N-oxide; eGFR= Estimated glomerular filtration rate.

© 2021 Lemaitre RN et al. *JAMA Network Open*.

eTable 4. Association of Serial Measures of Plasma TMAO and Related Metabolites With Incident Type 2 Diabetes Among 4442 Participants in the Cardiovascular Health Study With Follow-up Restricted to the First 12 Years

	-------Multivariable Model---------			--Multivariable Model with eGFR--		
	HR[1] (95% CI)		*P* value for trend	HR (95% CI)		*P* value for trend
**TMAO**						
Q1	1.00	- -	0.56	1.00	- -	0.63
Q2	1.20	(0.90, 1.61)		1.20	(0.90, 1.61)	
Q3	1.15	(0.86, 1.53)		1.14	(0.85, 1.52)	
Q4	1.00	(0.74, 1.35)		1.00	(0.74, 1.35)	
Q5	1.22	(0.90, 1.64)		1.21	(0.88, 1.64)	
**Choline**						
Q1	1.00	- -	0.43	1.00	- -	0.34
Q2	1.07	(0.79, 1.45)		1.06	(0.79, 1.44)	
Q3	1.00	(0.74, 1.34)		0.99	(0.73, 1.33)	
Q4	0.87	(0.64, 1.19)		0.86	(0.63, 1.17)	
Q5	0.96	(0.71, 1.30)		0.94	(0.69, 1.28)	
**Betaine**						
Q1	1.00	- -	0.03	1.00	- -	0.03
Q2	1.29	(0.98, 1.69)		1.29	(0.98, 1.70)	
Q3	1.08	(0.81, 1.45)		1.08	(0.81, 1.45)	
Q4	0.79	(0.57, 1.08)		0.79	(0.57, 1.08)	
Q5	0.86	(0.63, 1.19)		0.86	(0.63, 1.19)	
**Carnitine**						
Q1	1.00	- -	0.36	1.00	- -	0.38
Q2	1.23	(0.91, 1.66)		1.23	(0.91, 1.66)	
Q3	1.16	(0.86, 1.56)		1.16	(0.86, 1.56)	
Q4	1.27	(0.95, 1.71)		1.27	(0.94, 1.70)	
Q5	1.15	(0.85, 1.56)		1.15	(0.84, 1.55)	
**γ-Butyrobetaine**						
Q1	1.00	- -	0.24	1.00	- -	0.16
Q2	0.77	(0.58, 1.02)		0.76	(0.58, 1.01)	
Q3	0.67	(0.50, 0.90)		0.66	(0.49, 0.88)	
Q4	0.82	(0.61, 1.09)		0.79	(0.59, 1.06)	
Q5	0.80	(0.58, 1.09)		0.76	(0.54, 1.07)	
**Crotonobetaine**						
Q1	1.00	- -	0.63	1.00	- -	0.70
Q2	1.04	(0.75, 1.44)		1.04	(0.75, 1.44)	
Q3	0.90	(0.70, 1.17)		0.90	(0.69, 1.17)	
Q4	1.01	(0.77, 1.33)		1.01	(0.77, 1.33)	

© 2021 Lemaitre RN et al. *JAMA Network Open*.

Q5	1.10	(0.84, 1.44)		1.09	(0.83, 1.44)	

[1] Hazard Ratios of incident diabetes comparing each of the four higher metabolite quintiles to the lowest (reference) quintile, obtained from Cox regression models with serial measures of metabolite and time-updated covariates. The Multivariable Model adjusted for age, sex, race, site, education, income, BMI, waist, smoking, physical activity, systolic blood pressure, hypertension, LDL, CHD, animal sourced foods consumption, total energy intake. The Multivariable Model with eGFR additionally adjusted for eGFR. Abbreviations: HR=Hazard ratio; TMAO = Trimethylamine-N-oxide; eGFR= Estimated glomerular filtration rate.

© 2021 Lemaitre RN et al. *JAMA Network Open*.

eTable 5. Tests of Interaction Between the Metabolites and Age, Sex, BMI, Coronary Heart Disease, eGFR, and Dietary Intake of Animal-Sourced Foods in the Analysis of Incident Type 2 Diabetes

Effect modifier →	Median age	Sex	Median BMI	Prevalent CHD	eGFR (60 or more vs <60	Median consumption of animal sourced foods
TMAO	0.41	0.68	0.05	0.86	0.98	0.11
Choline	0.13	0.37	0.66	0.64	0.88	0.87
Betaine	0.71	0.04	0.003	0.43	0.03	0.29
Carnitine	0.37	0.41	0.17	0.52	0.72	0.06
γ-Butyrobetaine	0.45	0.53	0.61	0.77	0.81	0.74
Crotonobetaine	0.96	0.93	0.31	0.07	0.34	0.60

Each cell in the table corresponds to a separate Cox proportional-hazards regression analysis of incident type 2 diabetes that included the metabolite (linear variable), covariates and a multiplicative term between the metabolite (linear variable) and the effect modifier. Listed in the table are p-values for the multiplicative terms. The covariates included age, sex, race, site, education, income, BMI, waist, smoking, physical activity, systolic blood pressure, hypertension, LDL, CHD, animal sourced foods consumption and total energy intake. The pre-specified threshold of significance for these interaction tests was 0.0014 (0.05/36 tests).

© 2021 Lemaitre RN et al. *JAMA Network Open*.
